# Role of Gastrografin in Patients With Small Bowel Obstruction

**DOI:** 10.7759/cureus.9695

**Published:** 2020-08-12

**Authors:** Ibrahim Almafreji, Ugochukwu Chinaka, Amir Hussain, Mark Lynch, Richard Cottrell

**Affiliations:** 1 General Surgery, University Hospital Ayr, Ayr, GBR; 2 Pharmacy, University Hospital Ayr, Ayr, GBR

**Keywords:** gastrografin, small bowel obstruction, adhesional, post-operative ileus, constipation, general surgery

## Abstract

Introduction

Gastrografin (GGF) is a radiopaque contrast medium commonly used for diagnostic examination of the gastrointestinal (GI) tract. Available evidence suggests it has therapeutic and predictive value in the management of adhesional small bowel obstruction (ASBO). Thus, we investigated the use of GGF amongst patients who had a small bowel obstruction and audited the practice in University Hospital, Ayr.

Methods

Initial retrospective data of patients who had gastrografin for small bowel obstruction were extracted from April 2015 to August 2019 and analysed. After our local presentation and on implementing a GGF protocol, we prospectively collected data from February to June 2020 to close our audit.

Results

GGF showed a comparable therapeutic effect on ASBO in both audit cycles (72.2%-66.7%). Approximately 50% of unresolved cases were operated within 24 hours of GGF administration in both cycles. GGF consistently demonstrated a therapeutic benefit in refractory faecal impaction (100% in both cycles) and postoperative ileus (≥ 80%). Early use of computed tomography (CT) (less than 24 hrs) did not confer any added advantage (82.5% v 61.5%), however, it helped in making an appropriate diagnosis and the subsequent early gastrografin usage (78.3% v 92.3%) in ASBO.

Conclusion

GGF serves a very good therapeutic purpose in resolving ASBO, refractory constipation, and in rare non-resolving cases of postoperative ileus. Early CT diagnosis of ASBO is advocated before the administration of gastrografin. Unsuccessful resolution after 24 hrs of GGF is an indication for operative intervention.

## Introduction

Gastrografin (GGF) is a radiopaque contrast medium indicated for the radiographic examination of segments of the gastrointestinal (GI) tract (oesophagus, stomach, proximal small intestine, and colon) [1]. It is an iodinated hyper-osmolar agent being increasingly used to establish which patients will require an operation after the conservative management of non-resolving adhesional small bowel obstruction (ASBO) [1-2]. Surgery is usually indicated after the unsuccessful use of GGF [2].

Adhesive small bowel obstruction (ASBO) is the most common aetiology of small bowel obstruction. Other causes include abdominal wall hernia and small bowel malignancy. It presents mostly as a surgical emergency and is associated with high morbidity and, in some cases, mortality [2-3]. The adhesions causing such bowel obstructions are typically the footprints of previous abdominal surgical procedures [4].

The UK’s national audit of small bowel obstruction recommends the early use of computer tomography (CT) scanning for both diagnostic and prognostic information. They also recommend early GGF use in patients with ASBO who do not require emergency surgery. Surgery is recommended within 72 hours if initial management is unsuccessful [2].

The early use of GGF has also been advocated by the Bologna guidelines. This is in addition to other measures such as ensuring nil per os, intravenous fluids, and nasogastric tube decompression. Again, failure of conservative management within 72 hours should be operatively managed. Their proposed indications for immediate surgery included signs of bowel strangulation, bowel ischemia, and peritonitis [3].

In this audit, we sought to investigate the adherence to these guidelines for the management of ASBO in a district general hospital and the role of GGF in small bowel obstruction.

## Materials and methods

This audit was carried out in retrospective and prospective cycles. A retrospective analysis of the use of GGF at the University Hospital Ayr from April 2015 to August 2019 was performed. The data were derived from the online prescription program Hospital Electronic Prescribing and Medicines Administration (HEPMA). The sample size was determined by admissions with clear-cut small bowel obstruction (SBO) presentation to the surgical unit. Once patients were identified, additional information was extracted from patients’ case notes and online records. The results of this audit were presented to the surgical department at a local meeting. GGF was added as a HEPMA prescription rather than as a non-formulary one to enable the ease of prescription and audit. A GGF protocol was created and displayed around surgical wards as posters (see Appendix 1). We then prospectively reaudited patients who received GGF between February and June 2020. Results were analysed and compared to the preliminary audit.

Inclusion criteria

- Surgical patients presenting with small bowel obstruction.

- GGF used between April 2015 and August 2019.

Exclusion criteria

- Large bowel obstruction.

- Metastatic bowel obstruction (peritoneal carcinomatosis).

- GGF used for diagnostic purposes.

- No notes are available to assess the data points below.

We define the therapeutic use of GGF as contrast found in the colon on plain film imaging within 24 hours of its use and opening of the patient's bowels [3].

The results were divided into the following data points:

- Therapeutic rate of GGF in SBO and other associated conditions.

- Cases in which GGF was not effective, requiring operative intervention.

- Timing of CT scan.

- Timing of administration of GGF and operative interval when unsuccessful.

## Results

Retrospective audit

**Figure 1:**
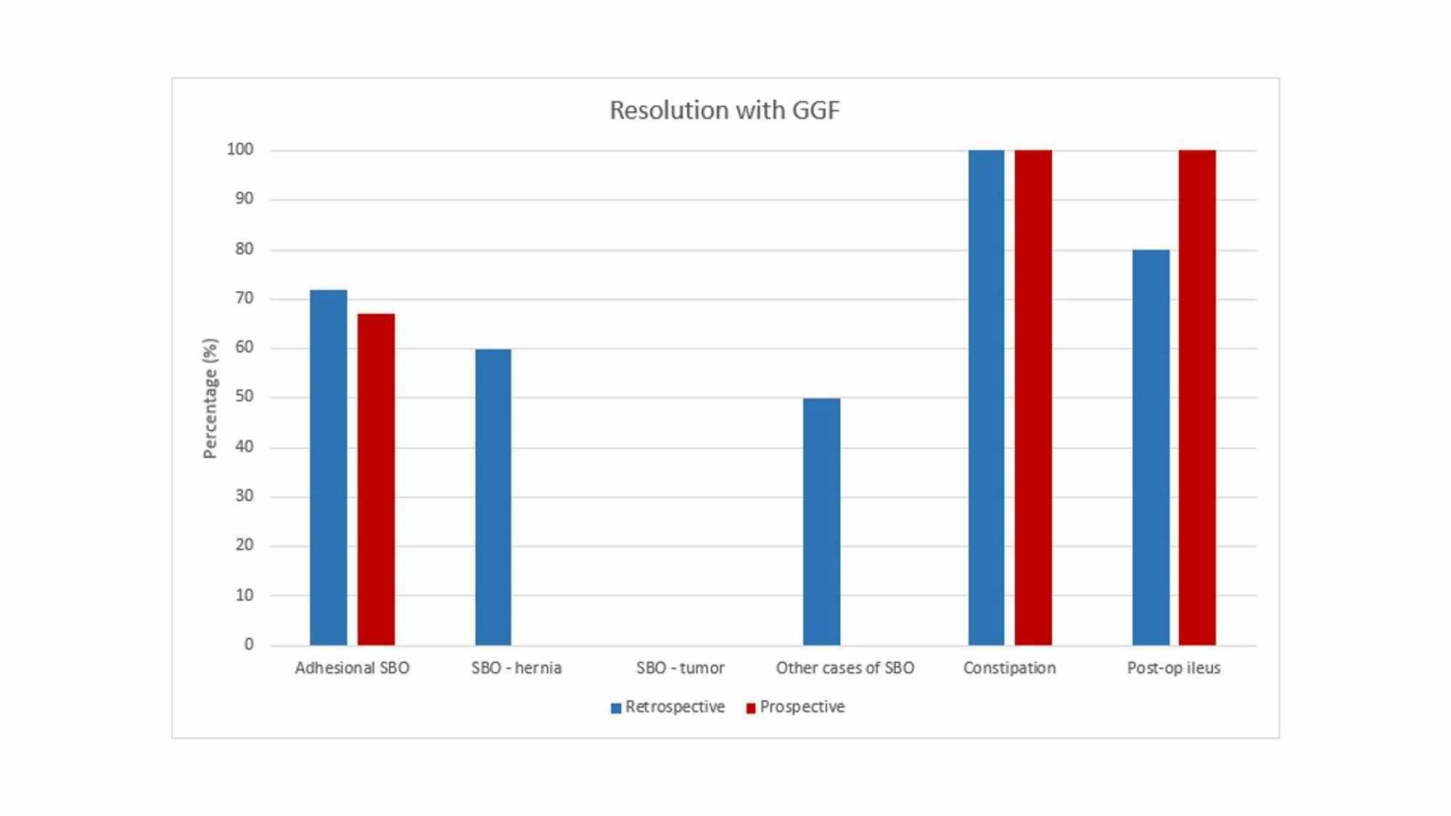
Resolution rates with gastrografin use

From a total of 61 patients who received GGF, 75.4% presented with SBO (n=46), 8.2% with postoperative ileus (n=5), and 4.9% with faecal impaction (n=3). Eleven point five per cent (11.5%) of cases were used for diagnostic purposes (n=7) and these were excluded from the analysis (Figure 1).

Seventy-eight point three per cent (78.3%) of those with SBO were due to adhesions (n=36). Gastrografin provided therapeutic benefit in 72.2% (n=26) of ASBO cases (Figure 1). Twenty-two point two per cent (22.2%) proceeded to surgical management (n=8) while 5.6% of the cases both did not respond to GGF and were not fit for surgery (n=2). Intraoperatively, 100% (n=8) of operated cases demonstrated adhesive bands and underwent adhesiolysis. Fifty per cent (50%; n=4) proceeded to theatre within 24 hours of gastrografin administration while 50% (n=4) went within 48 hours (Table 1).

**Table 1:** Results of small bowel obstruction cases only GGF: gastrografin

Criteria	Retrospective audit	Prospective audit
Surgery following unsuccessful GGF use	12/46 (26.1%)	4/13 (31%)
Surgery within 24 hours of unsuccessful GGF use	6/11 (54.5%)	2/4 (50%)
Surgery within 48 hours of unsuccessful GGF use	5/11 (45.5%)	0/4 (0%) 2/4 (50%) >72 hours
CT scan <24 hours	38/46 (82.6%)	8/13 (61.5%)
GGF <48 hours	36/46 (78.3%)	12/13 (92.3%)

Non-adhesional SBO contributed to 17.4% of SBO cases (n=8). These were further subdivided into SBO secondary to (a) hernias and (b) tumours. Those secondary to hernias made up 10.9% of the total SBO numbers (n=5). The specific location of the hernias was variable: one femoral, one umbilical, one incisional, and two parastomal. GGF was therapeutic in 60% of them (n=3), while 40% required an operation (n=2) (Figure 1). Operative findings showed an incarcerated umbilical and femoral hernia, respectively. The latter was operated on within 24 hours of GGF use while the former’s operative timing could not be confirmed (Table 1).

Six point five per cent (6.5%) of those with SBO were found to have tumours (n=3). None (0%) of these cases responded to GGF. Sixty-seven per cent (67%) of them proceeded to surgery (n=2) while the other patient was palliated. Fifty per cent (50%; n=1) was operated on within 24 hours of GGF administration and the other half within 48 hours (Table 1).

Three point three per cent (3.3%; n=2) of the SBO cases did not fit our classification. One case was a diagnosis of distal intestinal obstruction syndrome due to cystic fibrosis and the other case involved a CT scan diagnosis suggesting possible mesenteric volvulus. GGF, however, demonstrated a therapeutic effect in the latter.

GGF was found to be utilised in cases not involving SBO (13.1%, n=8). A few patients (4.9%, n=3) presented with faecal impaction, which did not resolve with various other laxatives. Hundred per cent (100%) of these cases resolved with GGF. Eighty per cent (80%; n=4) of patients with a postoperative ileus (n=5) resolved with GGF (Figure 1). No complications associated with GGF use were encountered.

A CT scan was performed within 24 hours in 82.6% of cases presenting with SBO (n=38). Additionally, GGF was given within 48 hours of admission/referral in 78.3% of the patients with SBO (n=36) (Table 1).

From the 46 patients of SBO, 26.1% (n=12) were taken to theatre. The time frame post-GGF administration could not be confirmed in one of the cases, as the operation date was not documented. Eleven cases provided us with a timeframe from GGF use to an operation. In six out of 11 (54.5%) cases, the decision to take the patient to theatre was made 24 hours after GGF use with no clinical improvement. In the other five patients (45.4%), that decision was made 48 hours post-GGF (Table 1). 

Prospective audit

The re-audit investigated 15 patients who received gastrografin for therapeutic purposes. Eighty-six point seven per cent (86.7%; n=13) of patients presented with SBO, all of which were adhesional in aetiology. GGF was utilised in a few other cases: faecal impaction (n=1) and postoperative ileus (n=1) (Figure 1).

In ASBO, fewer patients n=8 (66.7%) benefited from GGF use when comparing with our preliminary audit (72.2%) (Figure 1). A higher percentage of patients (31%, n=4) underwent operative management while fewer (n=1, 7.7%) were unfit for intervention in comparison to the initial audit (22% vs 5.6%, respectively). All the patients (n=4), in this cycle, had adhesiolysis from the intraoperative findings of adhesive SBO. The proportion of those undergoing surgery within 24 hours of GGF use was similar (n=2, 50%) when compared to the preceding audit. However, there was a clinical decision to delay surgical management (more than 72 hours after GGF use) in half (n=2) of those operated (Table 1). This is more delayed than the retrospective audit, as all patients proceeded to surgical management within 48 hours.

Compared to the preliminary audit, there appeared to be no significant change in GGF ability to resolve faecal impaction and postoperative ileus (100%, n=2) (Figure 1). Moreover, a smaller percentage of patients (61.5%, n=8) received a CT scan within 24 hours of admission as compared to the initial audit (82.6%). There was an increase in early GGF use (n=12, 92.3%) when compared to the first audit (n=36, 78.3%) (Table 1). Again, no complications due to GGF administration were reported.

Unlike in the retrospective audit, there was no GGF use in non-adhesional SBO secondary to hernias or tumours. Overall, 80% of patients (n=8) experienced a positive effect within 24 hours of GGF use, whereas 20% (n=2) experienced this within 48 hours. 

## Discussion

Oral water-soluble radiological contrast agents, most commonly diatrizoate meglumine and diatrizoate sodium (gastrografin (GGF)), are radiopaque contrast mediums indicated for the radiographic examination of segments of the gastrointestinal tract [1]. It is a highly osmolar iodinated contrast agent and possesses a mild laxative effect with an osmolarity of 1900 mOsm. GGF is a hypertonic solution that causes the fluid to be drawn into the lumen, helping to reduce intestinal wall oedema, and stimulates peristalsis [5]. It is commonly used for diagnostic purposes, however, it is increasingly being utilised in the conservative management of ASBO [1-2]. SBO is a common acute surgical presentation, with a variety of causes such as adhesions, hernias, and tumours. Postoperative adhesions contribute to the majority of cases of SBO [2-4]. 

Our first aim was to demonstrate the therapeutic effect of GGF in SBO. From our study, more than 65 % of cases of SBO resolved with GGF administration demonstrating a significant benefit in conservative management of ASBO. This benefit is supported by evidence from randomised controlled trials that detailed further benefits, as providing expedited resolution of obstruction, allowing earlier discharge and reducing the need for surgery in some patients [6-9].

The NASBO report identified many clinicians using GGF in the presence of hernias and tumours [2]. We noted such usage in our earlier audit cycle. However, there’s no literature supporting this indication. The audit provided the opportunity to address this in our service. The closed-loop cycle did not reveal any further practice.

All cases of faecal impaction (100% - both cycles) and ileus (100% - closed-loop) resolved with GGF in our findings. This is in agreement with the available literature on the resolution of faecal impaction and the shortening of the duration of postoperative ileus with GGF. Gu L et al. in a randomised clinical trial concluded that gastrografin was more effective than enemas in the treatment of faecal impaction [10], while Vather R et al. reported an acceleration in time to flatus or stool and time to resolution of abdominal distention in patients with post-operative ileus [11]. GGF is certainly worth considering when managing post-operative ileus and refractory constipation.

Investigating SBO is based on plain film and CT imaging to reach a diagnosis and produce an appropriate management plan [2]. CT scans are highly specific in diagnosing SBO [12]. As per NASBO guidelines, we analysed the early use of CT imaging [2]. There was a decrease in the use of CT scanning within 24 hours when comparing to the preliminary audit (82.5% v 61.5%). This may be attributed to the COVID-19 pandemic during which the prospective audit was performed. Nevertheless, a CT scan is recommended when suspecting SBO.

NASBO standards recommend GGF administration within 48 hours of admission for ASBO [3]. Results showed 78.3% of cases received GGF within 48 hours in the initial audit, whilst 92.3% was seen in the closed-loop. This showed an increase in early use. The early use of GGF allows us to predict whether the SBO will resolve. Consequently, we can decide on timely operative management and reduce the risk of bowel ischemia and length of hospital stay.

Bologna recommendations provide evidence to show that surgery should be considered if obstruction doesn’t resolve after >72 hours of non-operative management [13]. Our retrospective audit demonstrated that 45.5% of patients went to theatre within 24 hours of GGF administration while 54.5% were within 48 hours. Similarly in the prospective audit, 50% proceeded to surgery within 24 hours and the other half were after 72 hours. There was no significant difference in results 24 and 48 hours after GGF usage. Therefore, surgeons should consider surgery after 24 hours of unsuccessful GGF use.

## Conclusions

Gastrografin has proven to be significantly therapeutic in ASBO, refractory constipation, and rare cases of non-resolving postoperative ileus. It provides predictive value for the unsuccessful resolution of ASBO. Operative intervention is required within 24 hours of ineffective resolution from GGF. To expedite diagnosis and effective management, an early CT scan is mandatory. The use of GGF for incarcerated hernias and tumours is discouraged. The results of our findings and necessary policy changes were implemented in our hospital.

## Figures and Tables

**Figure 2:**
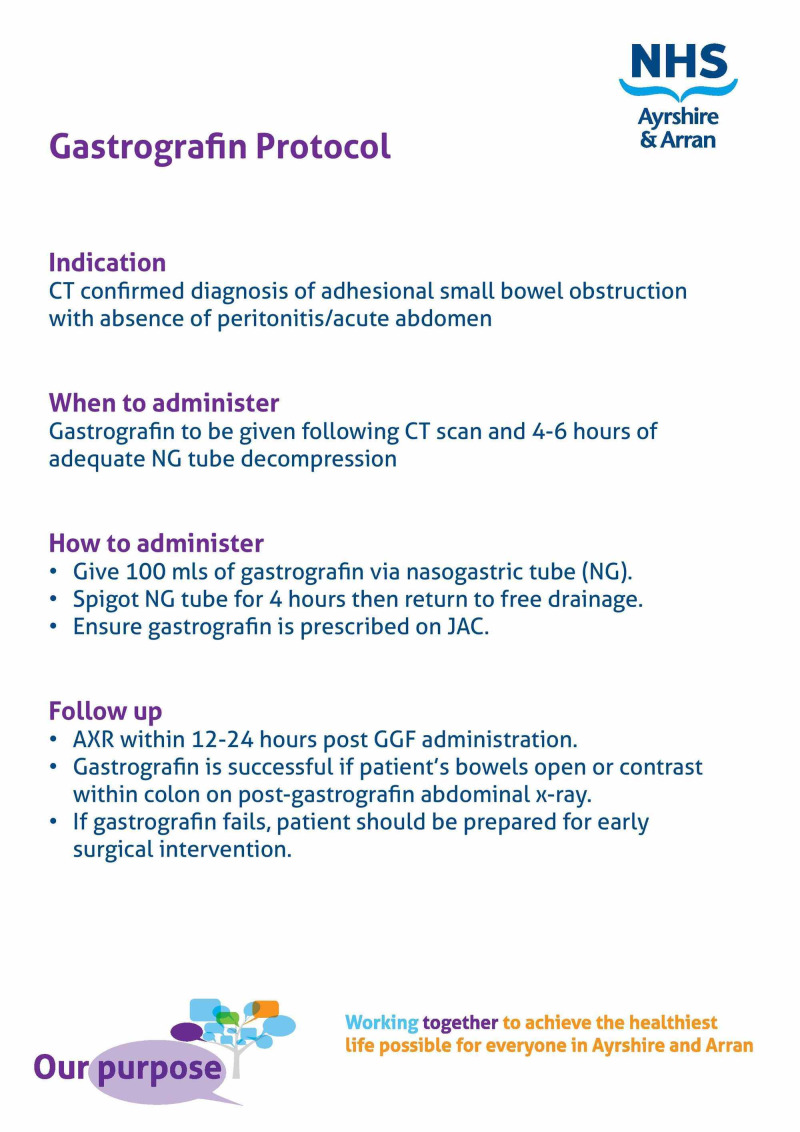
Gastrografin ward protocol

## Appendices

Appendix 1 
